# The contact-mediated response of peripheral-blood monocytes to preactivated T cells is suppressed by serum factors in rheumatoid arthritis

**DOI:** 10.1186/ar1804

**Published:** 2005-08-17

**Authors:** Manuela Rossol, Sylke Kaltenhäuser, Roger Scholz, Holm Häntzschel, Sunna Hauschildt, Ulf Wagner

**Affiliations:** 1 Department of Medicine IV, University of Leipzig, Leipzig, Germany; 2 Department of Orthopedics, University of Leipzig, Leipzig, Germany; 3 Department of Immunobiology, Institute of Zoology, University of Leipzig, Leipzig, Germany

## Abstract

Stimulation of monocytes/macrophages after cell contact with preactivated T cells has been suggested to contribute to the excessive TNF-α production in rheumatoid arthritis (RA). In this study, T cell-contact-dependent TNF-α production by peripheral-blood monocytes *in vitro *was investigated and found to be significantly lower in treated and untreated patients with RA than in healthy controls. This suppression was not due to a general deficiency of monocytes to respond, because responses to lipopolysaccharide were comparable in patients and controls. In agreement with the pivotal role of TNF-α in RA, T cell-dependent induction of TNF-α in synovial macrophages was fivefold to tenfold higher than in peripheral-blood monocytes from either patients or controls. The decreased response of peripheral-blood monocytes from patients with RA was found to be mediated by inhibitory serum factors, because the addition of patient sera to monocytes from healthy controls suppressed TNF-α response in the co-culture assay. Preincubation of monocytes from healthy controls with RA serum was sufficient to suppress the subsequent TNF-α response in T cell co-cultures, indicating that inhibitory factors do indeed bind to monocyte surfaces, which might represent a regulatory counter-action of the immune system to the long-standing and consuming autoimmune process in RA. There are some indications that apolipoprotein A-1 might be part of this regulatory system.

## Introduction

Cytokine production by monocytes/macrophages at sites of active inflammation is an important mechanism in the initiation and perpetuation of various chronic autoimmune diseases including type I diabetes mellitus, multiple sclerosis and rheumatoid arthritis (RA). The signals triggering this proinflammatory cytokine secretion *in vivo *are not completely understood. Although bacterial endotoxins such as lipopolysaccharide (LPS) and other microbial products are major stimuli of monocyte activation in infectious diseases, they are not considered to be relevant stimuli in autoimmune settings. So far, the most powerful known pathway inducing monocyte cytokine secretion *in vivo *in non-infectious situations is the direct cellular interaction with preactivated T cells [1].

The cell surface molecules involved in this T cell-dependent monocyte activation have been extensively investigated. T cell surface molecules, some of them upregulated on activation, such as CD69 [2,3], CD23 [4,5], integrins [6], CD40-CD40 ligand [7], LAG-3 [8], CD45 [9], LFA-1 and ICAM-1 [10] and membrane-bound cytokines [11] have all been implicated in this activation.

In RA, elevated levels of monocytic cytokines such as tumour necrosis factor (TNF)-α and IL-1β are present in the synovial membrane of diseased joints. Their relevance to disease pathogenesis has been highlighted by the clinical success of therapeutic strategies neutralizing TNF-α or IL-1β [12-14]. CD4^+ ^T cells, in contrast, have initially been implied in the pathogenesis of the disease because of the association of related HLA DRB1 alleles with susceptibility to and severity of the disease, and have subsequently been found to exhibit numerous pathological features such as oligoclonal expansions, contraction of T cell receptor repertoire, shortened telomere fragments indicative of increased replicative history, and distinct pathological phenotypes [15-19]. The traditionally described paucity of cytokines of T cell origin in the RA synovial membrane, which has been considered an argument against the involvement of T cells in the pathogenesis of the disease, has been put into perspective by the more recent recognition of high levels of IL-15 [20], IL-17 and, although at low levels, IFN-γ [21] in rheumatoid joints.

The monocytic production of TNF-α and IL-1 in RA synovial membranes seems to be independent of T cell cytokines. It has therefore been suggested that the direct interaction of activated T cells with monocytes, rather than the T cell-based production of cytokines, is a major stimulus of the excessive levels of TNF-α and IL-1β in RA. In addition, monocytes have been shown to be able to produce matrix metalloproteinases after contact with T helper type 2 (Th2) cells [22], which further implicates this interaction in the breakdown of extracellular matrix and subsequent joint destruction in RA.

To investigate cell-contact-mediated activation of monocytes by preactivated T cells, monocytic cell lines or monocytes from healthy donors have been primarily used in co-culture with T cells from healthy donors [8,11,23-26]. Disease-relevant CD4^+ ^T cells isolated from the synovial membrane of patients with RA were also used as stimulators and found to be potent inducers of cytokine production in monocytic THP-1 cells [27], in monocytes from healthy donors [10,28] and in mononuclear cells isolated from synovial membranes of patients with RA [29]. So far, peripheral-blood monocytes of patients with RA have not been analysed for T cell-dependent cytokine secretion, although the involvement of circulating monocytes in the disease process has been suggested [30-32]. Here we show that the T cell-dependent response of monocytes is suppressed in RA and that serum factors contribute to this inhibition, most probably by coating monocyte cell surfaces.

## Materials and methods

### Patients and controls

Twenty patients with RA as defined by the 1987 revised criteria of the American College of Rheumatology [33] were enrolled into the initial study. The study design was approved by the University of Leipzig's Ethics Committee, and informed consent was obtained from each patient before study enrolment. Sixteen of the patients had rheumatoid factor (RF) IgM seropositive disease, and 15 patients expressed the RA-associated shared epitope (on either a DRB1*01 or a DRB1*04 allele).

The median age of the patients was 59 years (range 22 to 74). For each patient, an age-matched control was selected from healthy volunteers. Clinical parameters documented at the time of presentation to the outpatient department included tender and swollen joint count, and concentrations of C-reactive protein (CRP) and of RF IgM.

Six patients had recent-onset RA (disease duration less than 2 years) and had not received therapy with disease-modifying anti-rheumatic drugs (DMARD) before inclusion in the study. The median disease duration for the 14 patients receiving DMARD was 9.5 years (interquartile range 5.0 to 11.0). Current treatment regimens included methotrexate alone (*n *= 5) or in combination with cyclosporine A (*n *= 1) or hydroxychloquine (*n *= 1), intramuscular gold injections (*n *= 1), leflunomide (*n *= 1) or TNF-α-blocking agents (*n *= 5).

For analysis of the T cell-dependent response of synovial macrophages, synovial biopsy specimens were obtained from six patients with RA and active synovitis who underwent synovectomy in the Department of Orthopedics at Leipzig University. Synovectomized joints were elbow joints (*n *= 2), metacarpophalangeal joints (*n *= 1), ankle joints (*n *= 2), knee joint (*n *= 1) and wrist (*n *= 1). Five of the patients had RF IgM-positive disease; the median CRP was 14.6 mg/l. Three of the patients received no immunosuppressive therapy, whereas two patients were treated with methotrexate and one was treated with etanercept.

To explore the influence of clinical parameters of the disease on inhibitory serum activity, two additional patient cohorts were recruited. Twenty patients with non-active disease (CRP below 10 mg/l and not more than swollen joints) were identified from a pre-existing institutional serum bank and compared with 20 patients with intermediate to high disease activity (mean CRP 35.6, median swollen joint count 14, median tender joint count 18). As control groups with systemic inflammatory disease, sera from 9 patients with ankylosing spondylitis and from 9 patients with psoriatic arthritis were used.

### Isolation of monocytes

Peripheral-blood mononuclear cells (PBMCs) were obtained by Ficoll^®^-Paque (Pharmacia Biotech, Freiburg, Germany) density-gradient centrifugation [34]. After repeated washing in PBS containing EDTA, monocytes were isolated by negative magnetic depletion with hapten-conjugated CD3, CD7, CD19, CD45RA, CD56 and anti-IgE antibodies (MACS; Miltenyi Biotech, Bergisch Gladbach, Germany) and a magnetic cell separator (MACS) in accordance with the manufacturer's protocol. The cell preparations were more than 95% monocytes as determined by morphology and immunofluorescence staining with a monoclonal antibody against CD14 (BL-M/G14).

To obtain larger amounts of monocytes, PBMCs were separated by counterflow centrifugation with the J6-MC elutriator system (Beckmann Instruments, Palo Alto, CA, USA) as described previously [35]. The cell preparations were more than 90% monocytes as determined by morphology and immunofluorescence staining with a monoclonal antibody against CD14 (BL-M/G14). In the co-culture assays described below, the response of monocytes separated by this technique was indistinguishable from that of monocytes obtained by immunomagnetic separation (data not shown).

### Separation of human synovial macrophages from patients with RA

Synovial tissue specimens were cut into 2 to 4 mm^3 ^pieces and washed once in complete medium (RPMI 1640, 10% FCS, 200 μM L-glutamine, 100 U/ml penicillin, 100 μg/ml streptomycin). Then, 1 cm^3 ^of tissue was incubated in 10 ml of digestion solution (0.05 M HEPES buffer, 3 mg/ml type 1A collagenase, 1 mg/ml hyaluronidase, 0.1 mg/ml type IV deoxyribonuclease I in RPMI 1640) at 37°C for 30 to 45 min [36]. Tissue residues were removed, and the resulting single cell suspension was washed twice.

Synovial macrophages were isolated by positive magnetic separation with CD14-conjugated magnetic beads (MACS; Miltenyi Biotech) and a magnetic cell separator (MACS) in accordance with the manufacturer's protocol.

### Separation and stimulation of human T cells

Human T cells were isolated by counterflow elutriation from PBMC as described above. T cells (10^6^/ml) were cultured in RPMI 1640 supplemented with 5% human AB serum, 2 mM glutamine, 100 U/ml penicillin and 100 μg/ml streptomycin in culture flasks (Techno Plastic Products AG, Trasadingen, Switzerland) at 37°C and 5% CO_2_. To stimulate T cells, culture flasks were coated with 3.3 μg/ml monoclonal anti-CD3ε antibodies (R & D Systems Inc., Minneapolis, MN, USA) and soluble monoclonal anti-CD28 antibodies (BD Biosciences Pharmingen, San Diego, CA, USA) were added to the medium at a concentration of 0.8 μg/ml. After stimulation and incubation for 2 days, the cultures contained more than 90% CD3^+ ^T cells as determined by flow cytometric analysis with a monoclonal antibody against CD3. Cells were then washed three times with PBS, fixed for 1 min with 0.05% glutaraldehyde [11] and washed again three times with PBS. Fixed T cells were stored for up to 2 weeks at 4°C.

This method of cell fixation was shown to inhibit blast transformation and TNF-α and IL-2 production in response to phorbol 12-myristate 13-acetate and ionomycin (data not shown).

### Stimulation of human monocytes with T cells

Monocytes (1.5 × 10^6^/ml) were cultured in RPMI 1640 supplemented with 10% FCS, 2 mM glutamine, 100 U/ml penicillin and 100 μg/ml streptomycin in 96-well culture plates (Techno Plastic Products AG) at 37°C and 5% CO_2_. Fixed T cells were added at a T cell : monocyte ratio of 7:1 (or any other indicated ratio) and cells were incubated together for 24 hours (or any other indicated time). LPS (*Escherichia coli *O55:B5, 100 ng/ml) was used as a positive control for monocyte cytokine production. In some experiments, semi-permeable Anopore membrane inserts (0.02 μm pore size; Nunc Life Technologies) were fitted into the culture wells to separate the monocytes (lower chamber) physically from the T cells (upper chamber). After incubation, supernatants (200 μl per well, two wells per condition) were harvested and stored at -140°C until cytokine concentration was determined.

Data presented in this work show interactions of T cells with monocytes from different donors. Similar results were obtained when T cells were incubated with autologous monocytes (data not shown).

### Analysis of inhibitory effects of serum samples in co-culture assays

In some cell–cell contact experiments, monocytes from healthy donors were incubated with human sera either from healthy individuals or from patients with RA. In these experiments, FCS was replaced by 10% heat-inactivated, heterologous human serum matched for blood types. Sera were incubated at 56°C for 30 min, at 70°C for 10 min or at 95°C for 2 min before their addition to the co-culture assay. When preincubation of monocytes (10^6^/ml) with human sera was performed, the monocytes were incubated in RPMI 1640 with 50% heat-inactivated (56°C for 30 minutes) serum at 20°C for 30 min. Monocytes were subsequently washed three times in PBS before proceeding to the standard co-culture assay as described above.

### Cytokine measurement

TNF-α concentrations were determined with a commercially available enzmye immunoassay (Beckman Coulter, Coulter-Immunotech, Krefeld, Germany) in accordance with the manufacturer's protocol.

### Apolipoprotein A-1 measurement

The serum concentration of apolipoprotein A-1 (ApoA-1) was determined by nephelometry by using a commercially available test kit (N antisera against human ApoA-1; Dade Behring, Liederbach, Germany).

### Statistical analysis

For statistical analysis, the software package Sigma Stat (SPSS Inc., Chicago, IL, USA) was used. Before all comparisons, a normality test (Kolmogorov–Smirnov test with Lilliefors' correction) was performed. Student's *t*-test or the Mann–Whitney rank sum test were used for comparisons where appropriate. To compare cytokine production in the patient population with that of age-matched controls, pairwise comparisons were performed.

## Results

### T cell-dependent induction of TNF-α production in monocytes

To explore the requirements and dynamics of T cell-dependent monocyte stimulation, an *in vitro *co-culture system using glutaraldehyde-fixed T cells as stimulator cells was established as described previously [11]. As a positive control, LPS, a potent stimulator of monocytes, was used in all experiments.

**Figure 1:**
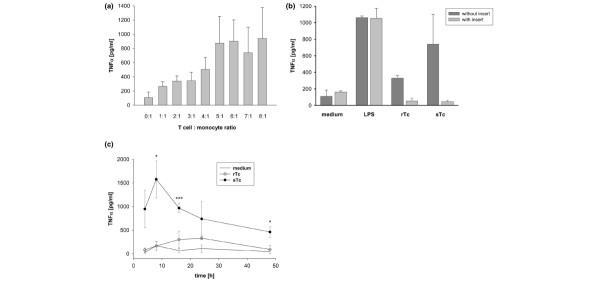
T cell induced production of TNF-α by monocytes from healthy donors. **(a) **Fixed stimulated T cells induce tumour necrosis factor (TNF)-α production by monocytes in a dose-dependent manner. Peripheral-blood T cells (10^6^/ml) were cultured for 48 hours in the presence of immobilized anti-CD3 (3.3 μg/ml) and soluble anti-CD28 (0.8 μg/ml) antibodies. Stimulated T cells were fixed and incubated for 24 hours with freshly isolated monocytes (1.5 × 10^6^/ml) at the indicated ratios. Values are means ± SEM from four different experiments. **(b) **Cell–cell contact is necessary for T cell-induced production of TNF-α in monocytes. Peripheral-blood T cells (10^6^/ml) were cultured for 48 hours in the presence or absence of immobilized anti-CD3 (3.3 μg/ml) and soluble anti-CD28 (0.8 μg/ml) antibodies. Stimulated (sTc) and resting (rTc) T cells were fixed and incubated for 24 hours with freshly isolated monocytes (1.5 × 10^6^/ml) at a ratio of 7:1 in a transwell system as described in the Materials and methods section. T cells and monocytes were physically separated by the semi-permeable membrane (with insert) or had direct cell–cell contact (without insert). Lipopolysaccharide (LPS; 100 ng/ml) was used as a positive control. Values are means ± SEM for experiments with three different donors. **(c) **T cell-induced TNF-α production by monocytes is time-dependent. Peripheral-blood T cells (10^6^/ml) were cultured for 48 hours in the presence or absence of immobilized anti-CD3 (3.3 μg/ml) and soluble anti-CD28 (0.8 μg/ml) antibodies. Stimulated (sTc) and resting (rTc) T cells were fixed and incubated for the indicated durations with freshly isolated monocytes (1.5 × 10^6^/ml) at a ratio of 7:1. Values are means ± SEM for experiments with three different donors. Levels of significance: * *P *< 0.05, *** *P *< 0.001.

The addition of fixed, heterologous T cells preactivated by immobilized anti-CD3 and anti-CD28 antibodies against CD14^+ ^peripheral-blood monocytes induced monocyte TNF production in a manner dependent on T cell concentration (Fig. 1a). Maximum stimulation of TNF-α production occurred at T cell : monocyte ratios between 5:1 and 8:1. Contact of monocytes with resting T cells even at the highest T cell : monocyte ratio did not lead to significant TNF-α production in those experiments (data not shown), and induced only a modest but not statistically significant increase in a later set of co-culture experiments (Fig. 1b). All subsequent experiments were performed at a T cell : monocyte ratio of 7:1, which was also used in several previously published studies with this experimental system [23,25,37]. To analyse the influence of the T cell origin on the monocyte response mediated by T cell contact, autologous and heterologous co-cultures were performed. Contact of monocytes with autologous or heterologous preactivated T cells led to the same amount of TNF-α (data not shown), so a heterologous co-culture system was used for subsequent experiments.

To exclude the influence of soluble mediators released by the fixed T cells, transwell experiments using a tissue culture plate insert with a microporous membrane with a pore size of 0.02 μm were performed. Monocytes placed in the bottom chamber of the transwell system, which had no physical contact with the prestimulated T cells present in the top chamber, failed to produce detectable amounts of TNF-α, indicating that cell-contact-dependent stimuli were necessary for monocyte activation (Fig. 1b). When the time course of monocyte TNF-α production after cell contact with preactivated T cells was analysed, a distinct kinetic profile comparable to that seen after stimulation with LPS was observed (Fig. 1c).

### T cell-induced TNF-α production of peripheral monocytes from patients with RA is decreased compared with TNF-α production in healthy donors

Although T cell–monocyte interaction has been proposed to contribute to the abundant TNF-α production seen in this disease, the role of peripheral monocytes from patients with RA in this interaction has not yet been investigated. To address this issue, CD14^+ ^cells were isolated from the peripheral circulation of patients with RA and from age-matched controls by negative immunomagnetic separation and were subsequently used in the co-culture assay.

**Figure 2:**
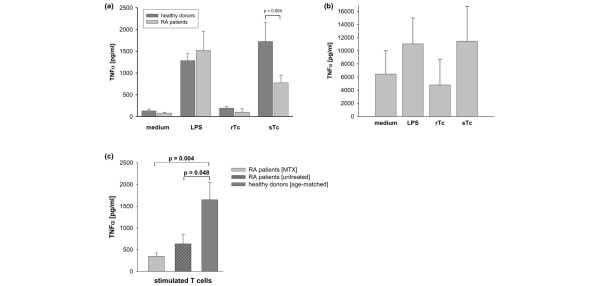
Decreased T cell induced production of TNF-α in monocytes from RA patients. **(a) **T cell-induced tumour necrosis factor (TNF)-α secretion by monocytes from patients with rheumatoid arthritis (RA) is decreased in comparison with those from healthy controls. Peripheral-blood T cells (10^6^/ml) were cultured for 48 hours in the presence or absence of immobilized anti-CD3 (3.3 μg/ml) and soluble anti-CD28 (0.8 μg/ml) antibodies. Stimulated (sTc) and resting (rTc) T cells were fixed and incubated with freshly isolated monocytes (1.5 × 10^6^/ml) at a ratio of 7:1. After 24 hours, the concentration of TNF-α was measured in the supernatant. Lipopolysaccharide (LPS; 100 ng/dl) was used as a positive control. Data are means ± SEM of values from 20 patients with RA and 20 age-matched controls. **(b) **Macrophages separated from the synovial membrane of patients with RA produce large amounts of TNF-α after contact with preactivated T cells. Stimulated (sTc) and resting (rTc) T cells were fixed and incubated with freshly isolated synovial macrophages from patients with RA (1.5 × 10^6^/ml) at a ratio of 7:1. After 24 hours, the concentration of TNF-α was measured in the supernatant. LPS (100 ng/ml) was used as a positive control. Data are means ± SEM of values from six independent experiments. Level of significance: not significant. **(c) **Decrease in T cell-induced TNF-α secretion by monocytes from patients with RA is independent of previous and current treatments. The graph depicts the T cell-induced TNF-α production by monocytes of patients with RA either receiving methotrexate (MTX; *n *= 5) or not receiving immunosuppressive treatment (untreated; *n *= 6). For comparison, results for six age-matched controls are given (*P *< 0.05, significant difference compared with untreated patients).

As seen in Fig. 2a, co-incubation of monocytes of patients with RA with preactivated fixed T cells resulted in significantly lower levels of TNF-α than in the controls. To exclude a global defect of monocyte cytokine production in RA, monocytes from patients with RA were stimulated with LPS as a positive control. In contrast to the T cell-induced TNF-α response, no difference in LPS-induced TNF-α production by monocytes was found between patients with RA and healthy controls.

In view of this unexpected finding, and with regard to the pivotal role of TNF-α in synovitic joints in RA, CD14^+ ^cells were separated with CD14^+ ^MicroBeads from synovial membrane biopsies from patients with RA, and tested for their capacity to produce TNF-α in the co-culture system. In contrast to the peripheral-blood monocytes from patients with RA, synovial mononuclear cells were found to be highly preactivated and to produce large amounts of TNF-α in the presence of resting T cells. In addition, they were found to increase their TNF-α production further after co-culture with preactivated fixed T cells and after stimulation with LPS (Fig. 2b). In parallel experiments, the influence of cell separation by CD14^+ ^MicroBeads and by negative immunomagnetic purification was compared. No significant differences between the two separation techniques with regard to the TNF-α response of synovial monocytes/macrophages were detectable. Most notably, however, the concentrations of TNF-α elicited in synovial monocytes/macrophages by T cell contact were fivefold to tenfold higher than those of peripheral-blood monocytes from either healthy donors or patients with RA under comparable experimental conditions. This result indicates that synovial cell populations are indeed likely to be the major source of the increased TNF-α load in RA, whereas peripheral monocytes are not.

To assess the influence of immunosuppressive therapy on T cell-dependent monocyte TNF-α production, patients were stratified into two groups: one of patients who were receiving DMARD therapy at the time of the study (*n *= 14) and one of patients with recent-onset RA who had not received steroid or DMARD medication before study enrolment (*n *= 6). T cell-dependent TNF-α production by peripheral-blood monocytes in the six patients with recent-onset RA was not significantly different (635 ± 210 pg/ml, *n *= 6) from that of patients who had received therapy (835 ± 233 pg/ml, *n *= 14).

When TNF-α production by monocytes from untreated patients with RA, who were age-matched with healthy controls, was analysed, monocytes from untreated patients produced significantly less TNF-α in a T cell-dependent manner (635 ± 210 pg/ml, *n *= 6) than those from controls (1,648 ± 398 pg/ml, *n *= 6, *P *= 0.048). Fig. 2c depicts the results from patients treated with methotrexate (*n *= 5), untreated patients with RA (*n *= 6) and age-matched healthy controls (*n *= 6).

Analysis of clinical and laboratory parameters of all patients tested did not reveal any association between T cell-dependent cytokine production and disease activity, RF status, immunogenetic markers, disease duration or the patient's age. Similarly, no age-dependent decline of cytokine production was observed in the age-matched control group.

### RA sera inhibit T cell-dependent TNF-α production by monocytes from healthy donors

Since monocytes from patients with RA were able to respond to LPS stimulation similarly to monocytes from healthy controls, we proposed that their diminished response in the co-culture assay was due to a disease-specific inhibitory mechanism present in the systemic circulation of patients with RA.

To test this hypothesis, serum exchange experiments were performed, in which monocytes from healthy donors were incubated with prestimulated T cells in the presence of 10% serum from either patients with RA or healthy controls. To avoid monocyte stimulation through blood type antigen-specific antibodies in the RA sera, patients and controls were matched for blood groups.

**Figure 3:**
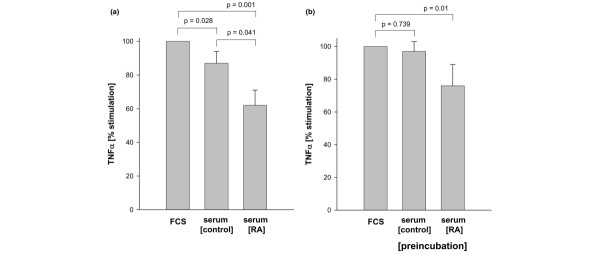
Sera from RA patients inhibit T cell-induced TNF-α production by monocytes from healthy donors. **(a) **Peripheral-blood T cells (10^6^/ml) were cultured for 48 hours in the presence of immobilized anti-CD3 (3.3 μg/ml) and soluble anti-CD28 (0.8 μg/ml) antibodies. The cells were fixed and incubated for 24 hours with freshly isolated monocytes (1.5 × 10^6^/ml) at a ratio of 7:1. The co-incubation assay was performed in the presence of 10% FCS, 10% serum from patients (serum [RA]) or from healthy donors (serum [control]). All data are expressed as percentages of TNF-α produced in the 10% FCS containing co-culture system (100%). Data are means ± SEM for 10 independent experiments; levels of significance are as indicated. **(b) **Monocytes from healthy donors were preincubated with 50% control sera (nine donors) or RA sera (six sera from patients with RA, which were previously found to inhibit T cell-dependent monocyte activation) and then washed thoroughly three times. Co-culture experiments were performed as described in the text in medium supplemented with 10% FCS. All data are expressed as percentages of TNF-α produced in the co-culture system containing 10% FCS. Data are means ± SEM for four independent experiments; levels of significance are as indicated.

The addition of sera from healthy controls to the co-cultures was found to inhibit T cell induced production of TNF-α when compared with control cultures containing culture medium supplemented with FCS (Fig. 3a). This is in line with previous observations reporting the inhibition of T cell-dependent monocyte activation by serum from healthy controls [25].

When sera from patients with RA were added, they also suppressed TNF-α production (Fig. 3a). In comparison with the control sera, this inhibition was found to be significantly stronger. A difference between RA sera and control sera was also seen in experiments in which monocytes were preincubated with either sera and then co-cultured with T cells in the standard FCS-supplemented culture medium (Fig. 3b). Whereas preincubation of monocytes in RA sera was found to induce the inhibitory effect, preincubation with healthy sera was not sufficient to influence TNF-α production (Fig. 3b). In these experiments, we used sera from patients with active RA that had previously been shown to exhibit a pronounced inhibitory activity.

**Figure 4:**
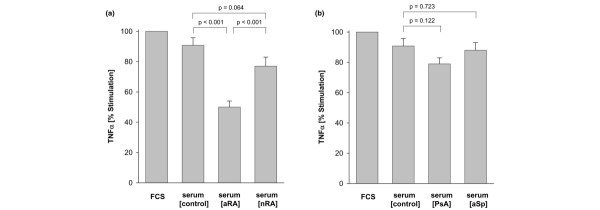
Inhibition of T cell-induced TNF-α production by monocytes is specific for active RA. **(a) **Preactivated peripheral-blood T cells (10^6^/ml; see Materials and methods) were fixed and incubated for 24 hours with freshly isolated peripheral-blood monocytes (1.5 × 10^6^/ml) at a ratio of 7:1. The co-incubation assay was performed in the presence of 10% FCS, 10% serum from healthy donors (serum [control], *n *= 10), from patients with active RA (serum [aRA], *n *= 20) or from patients with non-active RA (serum [nRA], *n *= 20). All data are expressed as percentages of TNF-α produced in the co-culture system containing 10% FCS. Data are means ± SEM; levels of significance are as indicated. **(b) **Co-incubation assays (see (a)) were performed in the presence of 10% FCS, 10% serum from healthy donors (serum [control], *n *= 10)), serum from patients with psoriatic arthritis (serum [PsA], *n *= 9) or from patients with ankylosing spondylitis (serum [aSp], *n *= 9). All data are expressed as percentages of TNF-α produced in the co-culture system containing 10% FCS. Data are means ± SEM; levels of significance are as indicated.

To analyse the influence of clinical parameters of disease activity on the inhibitory activity exhibited by the sera from patients with RA, sera from 20 patients with non-active disease were compared with sera from patients with high disease activity (for definition of active and non-active RA see the Patients and methods section). The results in Fig. 4a indicate that the inhibitory effect of RA sera is evident only in patients with active disease. To explore the disease specificity of this inhibitory activity further, sera from patients with ankylosing spondylitis and with psoriatic arthritis were used as two control groups with chronic, inflammatory autoimmune diseases. Figure 4b shows that sera from the disease controls did not inhibit monocyte cytokine production mediated by T cell contact and did not differ from those from healthy individuals.

**Figure 5:**
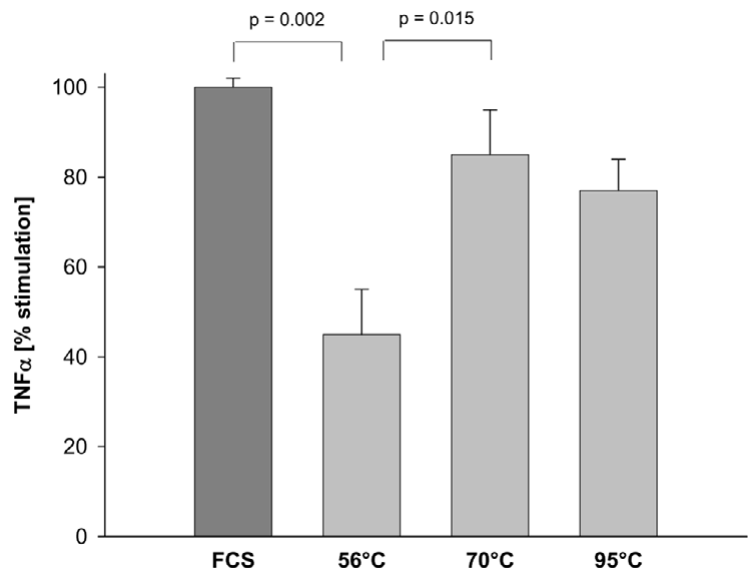
Inhibitory activity of rheumatoid arthritis (RA) sera is not heat stable. Six sera from patients with RA, which previously were found to strongly inhibit T cell-dependent monocyte activation were incubated at 56°C for 30 min, at 70°C for 10 min or at 95°C for 2 min. Co-culture experiments were performed in the presence of 10% FCS or 10% RA sera. All data are expressed as percentages of tumour necrosis factor-α produced in the co-culture system containing 10% FCS. Data are means ± SEM from six independent experiments. *P *= 0.002 (significant difference from the 100% values).

The selected RA sera with pronounced inhibitory activity, which were used in the preincubation experiments, were also used for experiments determining the heat resistance of the inhibitory activity. The sera were incubated at different temperatures and added to the standard co-culture assay. As seen in Fig. 5, heating the sera to 56°C (which was routinely used in the previous experiments) preserved the inhibitory activity, whereas increasing the temperature to 70 or 95°C resulted in a loss of that inhibitory activity. Thus, the inhibitory activity is due to heat-labile factors.

**Figure 6:**
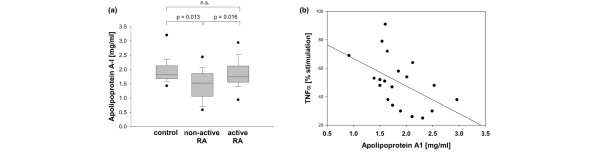
ApoA-1 concentrations are decreased in non-active RA but correlate with the inhibitory serum activity in active RA. **(a) **Box plot depicting ApoA-1 concentrations in sera from healthy controls (*n *= 20), in patients with active RA (*n *= 20) and in patients with non-active disease (*n *= 20). Levels of significance are given; n.s., not significant. **(b) **Scatter plot depicting the correlation between ApoA-1 concentrations and inhibitory serum activity in sera from patients with active RA. Each data point represents the ApoA-1 concentration in relation to the inhibitory activity. Tumour necrosis factor (TNF)-α production in the co-culture system containing 10% FCS is set to 100%. The regression line illustrates the negative correlation between the two parameters (correlation coefficient *R *= -0.527; *P *= 0.016).

Direct inhibition of T cell-dependent monocyte activation has been described previously for the serum protein ApoA-1. To analyse the contribution of ApoA-1 to the inhibitory activity found in sera from patients with RA, ApoA-1 concentrations were determined in sera from patients with active and non-active RA and from controls. The results depicted in Fig. 6a show that ApoA-1 concentrations in sera from patients with active RA are not different from those of the controls, but are significantly decreased in patients with non-active disease. However, when the inhibitory activity of each serum from patients with active disease was plotted against ApoA-1 concentrations, a significant correlation became apparent, because the inhibitory effect increased with increasing ApoA-1 concentration (correlation coefficient *R *= -0.527, *P *= 0.016; Fig. 6b). No correlation between ApoA-1 concentration and inhibitory activity was found in the sera from controls and from patients with non-active disease (data not shown).

## Discussion

Direct cell–cell contact of monocytes/macrophages with preactivated T lymphocytes leads to the secretion of high levels of proinflammatory cytokines and has been implied in the disturbed cytokine balance seen in RA [21,29,38]. As shown here and described previously, the most likely candidates responsible for the T cell-dependent TNF-α production are synovial macrophages from patients with RA. However, in some studies an involvement of peripheral-blood monocytes in the process of this chronic disease has also been suggested [30-32]. This raises the question of the contribution of monocytes to the increased TNF-α load seen in patients with RA. To address this we used monocytes from patients with RA in the co-culture system, because previous studies have examined monocytes from healthy donors only. The finding that monocytes from patients with RA produced less TNF-α than monocytes from controls was unexpected in view of the proposed contribution of this interaction to the excessive TNF-α levels observed in this disease. Monocytes from patients with RA and controls did not produce any cytokines in the absence of additional stimuli, indicating that peripheral-blood monocytes were not preactivated and that the cell separation techniques used did not lead to artificial *ex vivo *stimulation of the monocytes.

The induction of TNF-α unequivocally required direct cell contact of the monocytes with T cells, which excludes the possibility that the stimuli of cytokine production are soluble mediators released from the fixed T lymphocytes. Comparing the TNF-α levels produced by synovial monocytes/macrophages with those produced by peripheral monocytes clearly shows that monocytes are not major contributors to the TNF-α load in RA.

The experiments presented here also indicate that the reduced production of TNF-α is not an intrinsic feature of monocytes from patients with RA, because the monocytes are capable of a full TNF-α response to stimulation with LPS. This is in agreement with previous reports about the LPS response of monocytes from patients with RA [39,40], although the evidence is somewhat controversial [41,42]. Furthermore, when measuring other cytokines such as IL-1β, IL-8 and IL-10, we observed that monocytes from patients with RA responded equally well to LPS as did monocytes from controls (data not shown).

Because monocytes from patients with RA are fully capable of producing cytokines, the most likely explanation for the suppression of the T cell-dependent TNF-α response is the presence of regulatory serum factors. The serum protein ApoA-1 has been shown to act as a regulator of cytokine production [25]. The authors found that autologous serum from healthy controls was able to inhibit T cell-dependent TNF-α production in monocytes. They identified ApoA-1 as the molecule blocking the contact-mediated activation of monocytes. ApoA-1 is regarded as a 'negative' acute-phase protein and has been described as being present only in reduced levels in sera from patients with RA [43-45] and juvenile idiopathic arthritis [46], which makes it an unlikely candidate for systemic counter-regulation of cytokine production in RA. High levels of ApoA-1 have been found in the local synovitic environment in RA, where the molecule seems to act as an inhibitory regulator of cytokine production [47]. In its absence, one would expect cytokines to reach extremely high levels.

The present results confirm that patients with RA do not have an increased serum concentration of ApoA-1 compared with that of healthy controls. Consequently, the strong inhibitory activity of RA sera cannot be explained by an increased ApoA-1 concentration alone, although the result of a significant correlation between ApoA-1 concentration and inhibitory serum activity is remarkable.

The results are best explained by additional inhibitory factors that seem to be present in RA sera and seem to bind to the monocyte cell surface, as indicated by two indirect lines of evidence. First, monocytes from patients with RA cultured in FCS produced less TNF-α in response to activated T cells than those from controls (Fig. 4), which indicates that the soluble factor is transferred from the *in vivo *situation to the co-culture assay. The most likely mode of transfer of the factors would be in cell-bound form on the surface of the monocytes. Second, preincubation of monocytes from healthy controls in RA sera was sufficient to inhibit TNF-α production, and extensive washing did not abolish the inhibitory effect. Again, 'coating' of the monocyte surfaces by the suggested inhibitory factors, which prevents the full interaction between monocytes and T cells, might account for the reduced TNF-α production.

The inhibitory factor(s) described here seem to be specific for RA, and are most pronounced in patients with active disease. It can be proposed that, with increasing disease activity of RA, ApoA-1 (in addition to other factors) becomes upregulated and thus contributes to the downregulation of contact-mediated TNF-α production by monocytes.

## Conclusion

The data presented provide evidence for the existence of inhibitory, heat-labile factors in the serum of patients with RA, which downregulate the activation of peripheral-blood monocytes brought about by T cell contact. The possible physiological role of this regulatory mechanism and the specific molecules mediating the suppression remain to be determined.

## Abbreviations

ApoA-1 = apolipoprotein A-1; CRP = C-reactive protein; DMARD = disease-modifying anti-rheumatic drugs; FCS = fetal calf serum; IFN = interferon; IL = interleukin; LPS = lipopolysaccharide; PBMCs, peripheral-blood mononuclear cells; PBS = phosphate-buffered saline; RA = rheumatoid arthritis; RF = rheumatoid factor; TNF = tumour necrosis factor.

## Competing interests

The author(s) declare that they have no competing interests.

## Authors' contributions

MR was responsible for most of the experiments and data analysis as well as drafting the manuscript. SK and RS participated in the collection of the samples and in the interpretation of the results. HH supervised the collection of the samples as well as the design of the study. SH participated in the design of the study and in its coordination, and participated in the interpretation of the results. UW designed the study, participated in its coordination, participated in the interpretation of the results, and drafted the manuscript. All authors read and approved the final manuscript.
